# Carcinome épidermoïde de la vulve sur une grossesse gémellaire: à propos d’un cas aux cliniques universitaires de Lubumbashi

**DOI:** 10.11604/pamj.2013.14.70.2372

**Published:** 2013-02-19

**Authors:** Yves Isango Idi, Michel Manika Muteya, Christian Kakisingi Ngama, Roger Munan Mwazaz, Paul Ilunga Makinko, Fanny Malonga Kaj, Albert Mwembo Tambwe, Faustin Chenge Mukalenge

**Affiliations:** 1 Service de Gynécologie-Obstétrique, Cliniques Universitaires de Lubumbashi, Unilu, BP 1825, Congo; 2 Servive d’Anesthésie-Réanimation, Cliniques Universitaires de Lubumbashi, Unilu, BP 1825, Congo; 3 Service de Médecine Interne, Cliniques Universitaires de Lubumbashi, Unilu, BP 1825, Congo; 4 Service de Pédiatrie, Cliniques Universitaires de Lubumbashi, Unilu, BP 1825, Congo

**Keywords:** Cancer épidermoïde vulvaire, femme jeune, grossesse, prise en charge, Vulvar squamous cell carcinoma, young woman, pregnancy, management

## Abstract

Nous rapportons un cas de carcinome épidermoïde de la vulve chez gestante porteuse d’une grosse gémellaire qui a consulté dans le service de gynéco-obstetrique des Cliniques Universitaires de Lubumbashi à un stade avancé de la pathologie et après avoir essayé un traitement insuffisant ailleurs.

## Introduction

Le cancer vulvaire est une pathologie qui affecte essentiellement les patientes âgées de plus de 60 ans [1]. Il compte pour 3 à 5% des cancers gynécologiques [2]. Si 15% des cancers de la vulve sont diagnostiqués avant 40 ans, ils restent cependant exceptionnels pendant la grossesse où 16 cas ont été décrits pendant la grossesse [3]. On estime qu’en 2008, près de 454 cas de cancer de la vulve ont été diagnostiqués au Canada, et qu’environ 25 pour cent des femmes touchées mourront de ce cancer [4]. Nous rapportons un cas de cancer de la vulve sur une grossesse gémellaire au troisième trimestre, diagnostiqué à un stade tardif ainsi que les limites de la prise en charge en milieu sous équipé.

## Patient et observation

Il s’agit d’une gestante âgée de 32 ans porteuse d’une grossesse gémellaire, ayant 5 enfants en vie. Les accouchements antérieurs étaient eutociques à terme. Une laparotomie avait été réalisée pour une grossesse extra utérine rompue en 2010.

Une petite masse vulvaire prurigineuse avait été extirpée au mois de février 2011. Elle a débuté sa première consultation prénatale à 5 mois de grossesse (aout 2011), au cours de laquelle sera mis en évidence un nodule vulvaire d’apparition récente développée aux dépends de la grande lèvre droite, au niveau où avait été extirpée précédemment un nodule.

**Figure 1:**
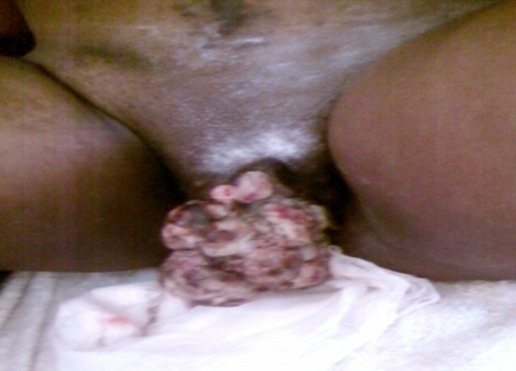
Masse vulvaire droite développée aux dépends de la grande lèvre droite et Adénopathie inguinale droite

Nous avons reçu la gestante deux mois après (octobre 2011), pour masse vulvaire de volume important saignant au contact. L’examen clinique avait révélé un bon état général, La grossesse gémellaire était âgée de 33 semaines et 2 jours et ne présentait aucune anomalie, une tumeur vulvaire de 15 centimètres de grand axe et 6 centimètres de petit axe (Figure 1), de surface irrégulière, saignant au contact, une adénopathie inguinale droite de 4 centimètres de diamètre.

**Figure 2:**
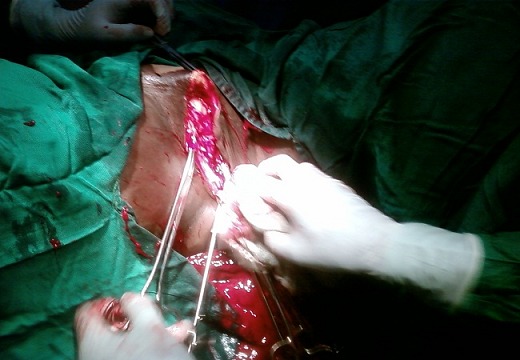
Hémivulvectomie droite. La tumeur a été extirpée en empotant 3 centimètres de tissu saint et adénectomie inguinale droite.

Nous avons conclu à une présomption de tumeur vulvaire maligne grade III selon la classification de la FIGO sur une grossesse gémellaire de 33 semaines et 2 jours. Une césarienne, une hémivulvéctomie droite ainsi qu’une adénectomie inguinale droite seront réalisées (Figure 2). L’examen anatomopathologique avait conclu à un carcinome épidermoïde peu différentié, la pièce d’adénectomie avait la même nature anatomopathologique que la tumeur primitive.

Une radiothérapie inguino-périnéale a été envisagée pour compléter la prise en charge mais elle ne sera pas réalisée à cause de l’inexistence d’un service de radiothérapie dans la ville et par manque de moyen financier pour un transfert vers un milieu équipé. L’évolution pot- opératoire immédiate sera marquée par la récurrence de la masse inguinale droite de consistance dure et la patiente décédera 4 mois âpres

## Discussion

Dans la grande majorité des cas, le cancer de la vulve touche la femme ménopausée de plus de 60 ans, survenant sur une muqueuse carencée en oestrogènes. Toutefois, certains papilloma virus humains (HPV 16) semblent jouer un rôle inducteur et peuvent constituer un risque, en particulier chez les femmes jeunes. Son origine dystrophique, la fréquence des lésions plurifocales, expliquent l′attitude thérapeutique classique, avant tout chirurgical large [5].

Il n′existe pas de test de dépistage, mais l′évaluation attentive de la vulve lors de l′examen gynécologique permettrait de découvrir la plupart des néoplasies vulvaires au stade pré envahissant. La biopsie de toute lésion suspecte est toutefois nécessaire pour éliminer un début d’envahissement [6].

L’évolution de la tumeur pendant la grossesse: la grossesse n’influence pas le l’évolution du cancer de la vulve. Pour GILLES BODY l’évolution des cancers vulvaires restent longtemps locorégionale et les métastases ganglionnaires sont précoces [7] Selon Andriew JM [5], la prise en charge est faite de la La vulvectomie totale avec évidement ganglionnaire inguinal bilatéral en monobloc est l′intervention de choix. Ce dernier peut ne pas être pratiqué dans le cas de petites lésions ou réduit à une simple adénectomie s′il existe un ganglion palpable.

Les autres interventions: tumorectomie large, hémivulvectomie, vulvectomie partielle antérieure ou postérieure, peuvent être pratiquées en fonction de l′âge et la présentation clinique. La chimiothérapie est parfois utilisée à titre palliatif pour une maladie évoluée ou métastatique, mais l′état général des malades comparé au faible taux de réponses, la rend rarement applicable. Par contre, plusieurs études en cours tentent d′évaluer l′intérêt de la chimiothérapie associée à la radiothérapie. Le rôle de cette chimiothérapie est plutôt celui d′une radiosensibilisation.

Nous avons réalisé une vulvéctomie partielle et une adénectomie dans un premier temps et dans un deuxième temps une radiothérapie a été envisagée mais non réalisée parce que n’existant pas dans notre milieu.

## Conclusion

Le cancer invasif de la vulve est très rare pendant la grossesse. Le cancer vulvaire chez la femme enceinte doit être traité de la même manière que chez une patiente non enceinte. Le traitement consiste en une vulvéctomie selon le contexte avec lymphadénectomie. En cas d’invasion ganglionnaire, une irradiation pelvienne est nécessaire et est malheureusement incompatible avec la poursuite de la grossesse. La voie d’accouchement doit être discutée cas par cas. L’accouchement peut entraîner un risque d’emboles vasculaires et de dissémination. Après une vulvéctomie, un accouchement par voie basse pourrait être autorisé (si cicatrisation correcte).
